# Inhibition of HSP90 as a Strategy to Radiosensitize Glioblastoma: Targeting the DNA Damage Response and Beyond

**DOI:** 10.3389/fonc.2021.612354

**Published:** 2021-03-17

**Authors:** Michael Orth, Valerie Albrecht, Karin Seidl, Linda Kinzel, Kristian Unger, Julia Hess, Lisa Kreutzer, Na Sun, Benjamin Stegen, Alexander Nieto, Jessica Maas, Nicolas Winssinger, Anna A. Friedl, Axel K. Walch, Claus Belka, Horst Zitzelsberger, Maximilian Niyazi, Kirsten Lauber

**Affiliations:** ^1^ Department of Radiation Oncology, University Hospital, LMU Munich, Munich, Germany; ^2^ Research Unit Radiation Cytogenetics, Helmholtz Center Munich, German Research Center for Environmental Health GmbH, Neuherberg, Germany; ^3^ Research Unit Analytical Pathology, Helmholtz Center Munich, German Research Center for Environmental Health GmbH, Neuherberg, Germany; ^4^ German Cancer Consortium, Munich, Germany; ^5^ German Cancer Research Center, Heidelberg, Germany; ^6^ Department of Organic Chemistry, NCCR Chemical Biology, University of Geneva, Geneva, Switzerland; ^7^ Clinical Cooperation Group Personalized Radiotherapy in Head and Neck Cancer, Helmholtz Center Munich, Neuherberg, Germany

**Keywords:** HSP90 inhibition, HSP90i, NW457, radiosensitization, glioblastoma, radiotherapy, hypermigration

## Abstract

Radiotherapy is an essential component of multi-modality treatment of glioblastoma (GBM). However, treatment failure and recurrence are frequent and give rise to the dismal prognosis of this aggressive type of primary brain tumor. A high level of inherent treatment resistance is considered to be the major underlying reason, stemming from constantly activated DNA damage response (DDR) mechanisms as a consequence of oncogene overexpression, persistent replicative stress, and other so far unknown reasons. The molecular chaperone heat shock protein 90 (HSP90) plays an important role in the establishment and maintenance of treatment resistance, since it crucially assists the folding and stabilization of various DDR regulators. Accordingly, inhibition of HSP90 represents a multi-target strategy to interfere with DDR function and to sensitize cancer cells to radiotherapy. Using NW457, a pochoxime-based HSP90 inhibitor with favorable brain pharmacokinetic profile, we show here that HSP90 inhibition at low concentrations with *per se* limited cytotoxicity leads to downregulation of various DNA damage response factors on the protein level, distinct transcriptomic alterations, impaired DNA damage repair, and reduced clonogenic survival in response to ionizing irradiation in glioblastoma cells *in vitro*. *In vivo*, HSP90 inhibition by NW457 improved the therapeutic outcome of fractionated CBCT-based irradiation in an orthotopic, syngeneic GBM mouse model, both in terms of tumor progression and survival. Nevertheless, in view of the promising *in vitro* results the *in vivo* efficacy was not as strong as expected, although apart from the radiosensitizing effects HSP90 inhibition also reduced irradiation-induced GBM cell migration and tumor invasiveness. Hence, our findings identify the combination of HSP90 inhibition and radiotherapy in principle as a promising strategy for GBM treatment whose performance needs to be further optimized by improved inhibitor substances, better formulations and/or administration routes, and fine-tuned treatment sequences.

## Introduction

Glioblastoma (GBM) is the most aggressive type of primary brain tumor with a highly dismal prognosis and a 5-year overall survival of less than 5% (1). Standard treatment involves maximal safe resection—if possible—followed by radio(chemo)therapy, and maintenance chemotherapy according to the EORTC/NCIC protocol (2–5). However, treatment failure and recurrence are frequent, and the major underlying reason appears to be the high level of inherent resistance against both chemo- and radiotherapy which represents a central hallmark of this cancer entity (6–9). Moreover, recent data indicate that the degree of radioresistance further increases during therapy—particularly when radiotherapy is applied in classically fractionated regimens (10, 11). Adaptive processes and an overt upregulation of the DNA damage response (DDR) have been reported to be crucial driving forces in this scenario (12–16). Since alternative fractionation regimens of radiotherapy have not shown relevant improvements (17–19), and recurrence frequently occurs within the irradiated volume (20), the question arises if biological targeting of the DDR can contribute to break GBM radiation resistance. Intriguingly, the DDR relies on high molecular weight proteins and multi-protein complexes which essentially require folding assistance and stabilization by chaperones, such as heat shock protein 90 (HSP90) (21–23). Thus, HSP90 actively contributes to radio- and chemoresistance of GBM and other cancer cells and represents an attractive target for biologically targeted radiosensitization, because HSP90 inhibition (HSP90i) —at least in principle—can affect multiple DDR pathways simultaneously (24–26). This was the focus of the present study for which we made use of the pochoxime-based, second generation HSP90 inhibitor NW457 with documented radiosensitizing potential in other cancer entities (27–32). We observed that diverse DDR regulators are overexpressed in human GBM cells and that their protein levels decrease upon HSP90i at low nanomolar doses which *per se* exhibited only limited cytotoxicity. In HSP90i-treated GBM cells, DNA damage repair was clearly impaired translating into significantly reduced clonogenic survival upon irradiation *in vitro. In vivo*, HSP90i augmented the therapeutic efficacy of fractionated, conebeam CT (CBCT)-based irradiation in an orthotopic GBM mouse model, although less potently than expected. Interestingly, the invasive morphology of radiotherapy-treated tumors was reverted by additional HSP90i, and *in vitro* migration analyses confirmed that HSP90i does reduce irradiation-induced GBM hypermigration.

## Materials and Methods

### Cell Lines and Reagents

The human GBM cell lines LN229 and T98G were obtained from ATCC (Manassas, VA, USA) and were cultured in Dulbecco’s Modified Eagle medium (DMEM), supplemented with 10% heat-inactivated fetal calf serum, 100 U/ml penicillin, and 0.1 mg/ml streptomycin (all from ThermoScientific, Schwerte, Germany) at 37°C and 7.5% CO_2_. The murine GBM cell line GL261 was obtained from the National Cancer Institute (NCI, Bethesda, MD, U.S.A.) and was cultured under same conditions. All cell lines were screened to be free from mycoplasma infection, and identity of human cell lines was confirmed by short tandem repeat (STR) typing (service provided by DSMZ, Braunschweig, Germany).

The HSP90 inhibitor NW457 (*epi*-pochoxime F) was previously described (27–31). For *in vitro* experiments, a 10 mM stock solution was prepared in DMSO (Sigma-Aldrich, Taufkirchen, Germany) and was further diluted to 100 µM with DMSO before final concentrations were adjusted in cell culture medium. Respective amounts of DMSO served as controls. For *in vivo* purposes, NW457 was dissolved at 100 mg/ml in DMSO and was further diluted in 0.9% NaCl (37°C), supplemented with 5% Tween-20 (all from Sigma-Aldrich). The vehicle formulation was used as control.

### X-Ray Treatment *In Vitro*

Irradiation of cells was done with an RS225 X-ray tube (200 kV and 10 mA, Thoreaus filter, 1 Gy in 63 s, Xstrahl, Camberley, Great Britain) as described (30).

### Quantitative Real-Time PCR (qRT-PCR)

Profiling of mRNA expression levels was performed by quantitative realtime RT-PCR as described (32). Briefly, total RNA was extracted from cells by NucleoSpin RNA II extraction kit (Macherey & Nagel, Dueren, Germany). 500 ng of isolated RNA were mixed with 5 µM random hexamers, 5 µM Oligo(dT)_18_, 500 µM dNTPs, 1 U/µl Ribolock RNase inhibitor, and 10 units/µl RevertAid transcriptase (all from ThermoScientific) and subjected to reverse transcription. Twenty nanograms of cDNA were employed for Realtime PCR runs with 300 nM primers in 1x Maxima SYBR Green qPCR Mastermix (ThermoScientific) on an LC480 qPCR platform (Roche Applied Science, Penzberg, Germany). Primer sequences are listed in **Supplementary Table 1**. Relative quantification was performed by the δδC_T_ method. Results were normalized to a matrix of reference genes comprising 18S rRNA, δ-Aminolaevulinate-synthase (ALAS), and β2-Microglobulin (B2M) and calibrated to the relative expression levels measured in primary human astrocytes (BioCat, Heidelberg, Germany). Three replicates were analyzed per cell line, and heatmaps were generated using the matrix visualization software Morpheus (https://software.broadinstitute.org/morpheus).

### Clonogenic Survival Assay

Clonogenic survival was assessed by colony formation assays as described before (30, 33). In brief, cells were detached by Trypsin/EDTA (ThermoScientific), counted with a Neubauer counting chamber, and seeded as single cell suspensions at defined numbers anticipating 20–100 colonies per well depending on the different irradiation doses into 6-well plates. Adherence was allowed for 4 h. LN229 and T98G cells were treated with 10 nM NW457 or DMSO for 24 h, irradiated, and incubated in the presence of 10 nM NW457 for 13 d. GL261 cells were treated in a similar fashion, except that NW457 was removed by medium exchange after the 24 h of pre-incubation. Colonies were stained with methylene blue (dissolved at 0.3% in 80% ethanol, both from Merck, Darmstadt, Germany), and all colonies containing more than 50 cells were counted under a Stemi 305 stereomicroscope (Carl Zeiss, Oberkochen, Germany). The percentages of surviving cells were determined and calibrated to the corresponding plating efficiencies. Regression was performed according to the linear-quadratic model.

### Viability Assay

Viability was determined by Alamar Blue assays (BioRad, Puchheim, Germany) as described (30). Briefly, 5,000 cells were seeded into 96-well plates, adherence was allowed for 4 h, and cells were treated with NW457 at the indicated doses. Upon incubation for 24–96 h, medium was replaced by fresh medium supplemented with 1/10 volume of Alamar Blue reagent, and Resazurin conversion was allowed at 37°C and 7.5% CO_2_ for 2–6 h. Resorufin fluorescence was measured on a Synergy Mx microplate reader platform (BioTek, Bad Friedrichshall, Germany), and results were calibrated to untreated controls.

### Quantitative Fluorescence Microscopy

DNA damage repair was examined by immunofluorescence staining of phosphorylated histone variant H2AX (γH2AX) and p53-binding protein 1 (53BP1), followed by quantitative fluorescence microscopy as described (32). Cells were seeded into 24-well plates supplemented with coverslips, allowed to adhere overnight, and treated with 10 nM NW457 or DMSO for 24 h before being irradiated at 2 Gy. At the indicated time points, cells were fixed with 3.7% isotonic paraformaldehyde (Merck), containing 0.1% Triton X-100 (v/v Sigma-Aldrich) for 10 min before being permeabilized with 0.5% isotonic Triton X-100 for 5 min. Unspecific binding sites were blocked with 3% isotonic bovine serum albumin (w/v, Sigma-Aldrich) and 0.1% Triton X-100 at 4°C overnight. Cells were stained with monoclonal mouse anti-γH2AX (Merck Millipore) and polyclonal rabbit anti-53BP1 (Bio-Techne, Wiesbaden, Germany) antibodies diluted in 3% isotonic bovine serum albumin and 0.1% Triton X-100 for 2 h at room temperature. After extensive washing with PBS plus 0.1% Triton X-100, cells were stained with Alexa488-coupled goat-anti-mouse IgG and Alexa568-coupled goat-anti-rabbit IgG (both from ThermoScientific) for 1 h. DNA was stained with 2 µg/ml Hoechst 33342 (Sigma Aldrich) for 10 min. Upon several washing steps with PBS plus 0.1% Triton X-100, coverslips were mounted with 4 µl mounting medium (Sigma-Aldrich) onto object slides. Microscopic analysis was performed with a Zeiss AxioObserver Z1 inverted microscope, equipped with an LCI Plan-Neofluar 63x/1.3 glycerol objective, an AxioCam MR Rev3 camera, and ZEN 2.3 software (all from Carl Zeiss). For image acquisition, 31 z-stacks with 250 nm interstack distance were captured, and deconvolution was performed with ZEN 2.3 software. For quantification of DNA damage repair, γH2AX/53BP1 double-positive foci were used. At least 20 nuclei of non-deformed morphology were selected for each condition, and foci were counted by hand. Results are depicted as individual data points with superimposed means and 95% confidence intervals.

### Live-Cell Microscopy of Cell Death Morphology

For live-cell imaging, a Zeiss AxioObserver Z1 inverted microscope, equipped with an AxioCam MR Rev3 camera, an XL multi S1 incubation chamber, and a PS1 compact heating unit (both from Pecon, Erbach, Germany) was used. Briefly, cells were seeded into Ibidi µ-slides (Ibidi GmbH, Martinsried, Germany) and allowed to adhere for 4 h before treatment with 10 nM NW457 or DMSO for 24 h and irradiation at 4 Gy. Live-cell imaging was initiated 1 h after irradiation and performed over 12 d. Images were captured in 12 min intervals, and movies were processed with Fiji software.

### Wound Healing Assay

Migration of GBM cells was assessed by wound healing assays. Cells were seeded into Ibidi µ-slides supplemented with culture inserts (both from Ibidi) and allowed to adhere overnight. Cells were treated with 30 nM NW457 or DMSO for 24 h, irradiated at 3 Gy, and live-cell imaging was performed for 12 h. Images were captured in intervals of 3 min, and cell migration was analyzed using the manual tracking plugin tool (ImageJ) as previously described (31, 34). Migration was quantified in form of colonized area (four regions of interest per condition in three independent experiments) and accumulated distance per cell over time (at least 25 randomly picked cells per condition).

### SDS-PAGE and Western Blot

Reducing gradient SDS-PAGE and western blot analyses of whole cell lysates (20–400 µg total protein per lane) were performed as described before (30, 35). Briefly, cells were lysed in lysis buffer (50 mM Tris-HCl pH 7.6, 150 mM NaCl, 1% Triton X-100 (v/v) (all from Sigma Aldrich), 1 x EDTA-free protease inhibitor cocktail (Roche)), protein concentrations were measured by Bradford assay (BioRad, Feldkirchen, Germany), and 20 - or 400 µg of total protein were subjected to gradient (4–15% or 6–15%) SDS-PAGE. Proteins were transferred onto PVDF Immobilon FL membranes (Merck Millipore), membranes were blocked with 5% low-fat milk powder (Carl Roth, Karlsruhe, Germany), dissolved in TBST buffer ((13 mM Tris-HCl pH 7.5, 150 mM NaCl, 0.02% Triton X-100 (v/v)), and incubated with primary antibodies at 4°C overnight. Primary antibodies used for western blot analyses were: Rabbit-anti-ATM, rabbit anti-ATR, rabbit-anti-FANCA, mouse-anti-RAD51 (Merck Millipore), mouse-anti-CHK1, mouse-anti-Vinculin, mouse-anti-α-tubulin (Sigma-Aldrich), rabbit-anti-KU70, rabbit-anti-XRCC3, rabbit-anti-MGMT (Biozol, Eching, Germany), mouse-anti-CHK2, mouse-anti-B-Raf (BD Transduction Laboratories, Heidelberg, Germany), rabbit-anti-RPA1, rabbit-anti-RBBP8 (Biomol, Hamburg, Germany), rabbit-anti-KU80, rabbit-anti-DNA2 (Thermo Scientific), rabbit-anti-p53 (Cell Signaling, Leiden, Netherlands), rabbit-anti-DNA-PKcs (Abcam, Berlin, Germany), mouse-anti-NHEJ1 (Santa Cruz, Heidelberg, Germany), rabbit-anti-LIG4 (Origene, Herford, Germany), and mouse-anti-HSP70 (BD Biosciences). Upon washing with TBST, membranes were incubated with IRDye800-conjugated secondary antibodies (LI-COR Biosciences, Bad Homburg, Germany) for 1 h at room temperature. Measurements and quantifications of IR800 dye fluorescence were performed with an ODYSSEY scanner (LI-COR Biosciences, Bad Homburg, Germany). Relative signal intensities were normalized to a matrix of vinculin and α-tubulin, calibrated to the untreated controls, and heatmaps were visualized using the matrix visualization software Morpheus (https://software.broadinstitute.org/morpheus).

### Transcriptome Analysis Via RNA Sequencing

Transcriptome profiling was performed by 3’-RNA sequencing. Prior to sequencing, RNA integrity was assessed using the Agilent Bioanalyzer RNA 6000 Nano Kit (Agilent Technologies, Waldbronn, Germany) by calculating the percentage of fragments > 200 nucleotides (DV200). Sequencing libraries were prepared with 100 ng total RNA using the QuantSeq 3’-RNA-Seq Library Prep Kit FWD for Illumina (Lexogen GmbH, Vienna, Austria) according to the manufacturer’s instructions for single-indexing and good RNA quality. For library amplification, PCR cycles were determined using the PCR Add-on Kit for Illumina (Lexogen), and the individual libraries were amplified with 17 PCR cycles. Quantity and quality of sequencing libraries were assessed using the Quanti-iT PicoGreen dsDNA Assay Kit (ThermoScientific) and the Bioanalyzer High Sensitivity DNA Analysis Kit (Agilent Technologies). Libraries were sequenced in 150 bp paired-end mode on a HiSeq4000 sequencer (Illumina, Berlin, Germany). The pool of individually barcoded libraries was distributed across the lanes of the same flow-cell aiming for approximately ten million paired-end reads per sample.

For sequence data processing, adapter sequences were removed using BBDUk (https://jgi.doe.gov/data-and-tools/bbtools). Human fastq-files including forward-reads were subjected to alignment against the human genome reference genome (GRCh38) using STAR (36). Aligned reads were quantified *via* htseq-count employing appropriate transcriptome gtf-files (37). FastQC was utilized for analyzing quality of unaligned and aligned reads (https://www.bioinformatics.babraham.ac.uk/projects/fastqc/) followed by summarization *via* multiQC (https://multiqc.info). Genes with a raw read count (for the whole dataset) smaller than five times the total number of samples were excluded. Correlation heatmaps were employed to analyze data consistency and technical outlier detection, and shrinked (apeglm) log2 expression values were determined (38). Calculation of differentially expressed genes and geneset enrichment analyses (GSEAs) were performed on the basis of log2 expression values (39). Reactome functional interaction (FI) networks were constructed and analyzed in Cytoscape (40, 41). iRegulon was employed to identify potential transcriptional regulators (42).

### Orthotopic Mouse Glioblastoma Model and Contrast-Enhanced, Conebeam CT-Based, Fractionated Radiotherapy

All animal experiments were performed in accordance with the FELASA guidelines and upon ethical approval by the *Regierung von Oberbayern*. Female C57BL/6 mice were obtained from Charles River (Sulzfeld, Germany), and housed in groups of maximally four animals in individually ventilated cages (GM500, Tecniplast, Hohenpeißenberg, Germany) in a specified, pathogen-free animal facility with a 12 h day/night cycle. Standard rodent feed (from Ssniff, Soest, Germany) and water were provided *ad libitum*. Animals were inspected on a daily basis and sacrificed when reaching pre-defined health scores. Criteria for immediate sacrifice comprised the following: Strongly altered hygiene behavior, flattened breathing, body weight loss of ≥ 20%, ulcerating wounds, epileptic seizures or spasms, paralysis of extremities, bloody diarrhea, apathy, hunchbacked posture, self-mutilation, isolation from the group. In milder occurrence of these criteria, mice were sacrificed within 48 h. Intracranial implantation of GL261 cells was performed as described recently (43). Briefly, mice were medicated with 200 µg/g metamizol (WDT, Garbsen, Germany) and anesthetized by intraperitoneal injection of 100 µg/g ketamine and 10 µg/g xylazine (both from WDT). Mouse heads were mounted onto a stereotaxic frame (David Instruments, Tujanga, CA, USA), skulls were exposed by longitudinal skin incision, and a hole was drilled 1.5 mm laterally (right) and 1 mm anteriorly to the bregma using a pair of 23G and 21G microlances (BD Biosciences). Then, 90,000 GL261 cells (in 1 µl PBS) were slowly injected into the right striatum, using a stereotactically guided syringe (Hamilton, Bonaduz, Switzerland). Once the syringe was withdrawn, skin was closed with Ethibond Excel 5-0 suture material (Ethicon, Norderstedt, Germany), and mice were monitored until regaining consciousness. Starting at d7 after implantation, tumor growth was monitored by contrast-enhanced, conebeam computed tomography (CBCT) scans twice weekly using the small animal radiation research platform (SARRP, X-Strahl, Camberley, Great Britain) (44). For acquisition of CBCT scans, 360 projection images were captured (1° per image, x-ray tube settings: 60 kV, 0.8 mA, 1.0 mm aluminium filter). To enhance the contrast of soft tissue, 300 μl Imeron-300 (Bracco, Konstanz, Germany) were administered intravenously before CBCT scanning. Irradiation was performed at a weekdaily fractionation regimen of 2 Gy (2× 5× 2 Gy in total) with two contralateral beams (gantry positions − 90° and 90°) and 3× 9 mm^2^ collimation (fixed nozzle, x-ray tube settings: 220 kV, 13 mA, 0.15mm copper filter) on d7-11 and d14–18. Isocenters were aligned to the centers of contrast enriching volumes, and treatment planning was executed with Muriplan software (X-Strahl). NW457 was administered intraperitoneally at 10 µg/g or 50 µg/g 24 h before irradiation. Tumor volumes were determined by Lx Wx H measurements of the 3 longest orthogonal axes as described (43), and 3D reconstructions were generated in 3D-Slicer (www.slicer.org/).

### Histological Analyses

For histological analyses, mice were anesthetized by intraperitoneal injection of 50 µg/g pentobarbital (WDT), followed by cardial perfusion with 3.5% paraformaldehyde (Sigma-Aldrich) as described (43). Then, brains were explanted and fixed with 3.5% paraformaldehyde for 48 h at 4°C. Brains were dehydrated for 48 h in 30% sucrose (Sigma-Aldrich), embedded in NEG-50 frozen section medium (ThermoScientific) and stored until analysis at −80°C. Slices of 40 µm thickness were prepared with a Microm HM355S microtom (ThermoScientific), stored in cryopreserving solution (200 mM Na_2_HPO_4_, 200 mM KH_2_PO_4_, 25% ethylenglycol (v/v), 25% glycerol (v/v) (all from Sigma Aldrich)) at −20°C, before being stained with Mayer’s hematoxylin and eosin (both from Merck) for 1 min each. After dehydration in 70, 96, and 100% ethanol, and xylene, slices were mounted onto microscope slides using Entellan (Merck). Microscopic analysis was performed on an AxioLab A.1 microscope, equipped with an AxioCam Erc5s camera and AxioVision 4.9 software (all from Carl Zeiss).

### Statistical Analyses

Statistical analyses were performed using OriginPro 9.1 software (OriginLab Ltd., Northhampton, MA, USA). Results are shown as individual data points of all replicates, means ± s.d., or means ± 95% confidence intervals as indicated. For group comparisons, two-sided Student’s *t*-tests or ANOVAs (one-way or two-way) were employed as indicated. Survival analyses were performed according to Kaplan-Meier with log-rank testing.

## Results

### HSP90 Inhibition by NW457 Leads to Downregulation of DNA Damage Response Factors on the Protein Level, Impaired DNA Damage Repair, and Reduced Clonogenic Survival in Response to Ionizing Irradiation in Glioblastoma Cells

**Figure 1:**
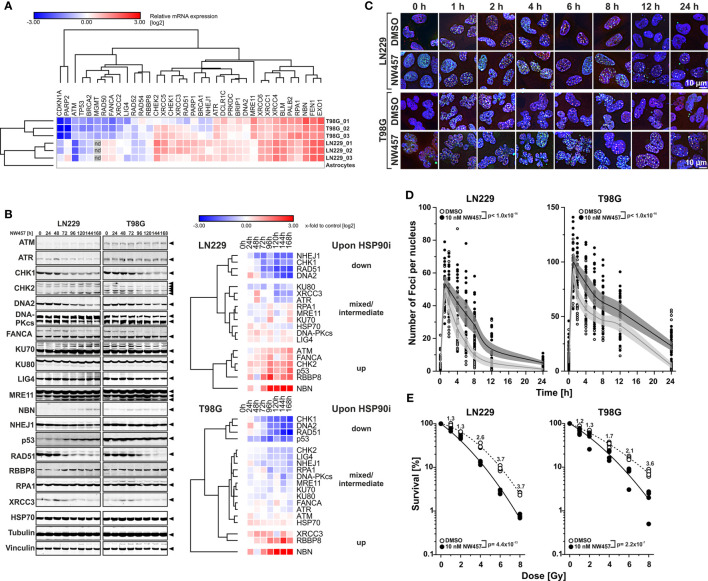
HSP90i by NW457 leads to downregulation of DNA damage response factors, impaired DNA damage repair, and reduced clonogenic survival in response to ionizing irradiation in human glioblastoma cells. **(A)** Transcriptomic profiling of regulators of the DNA damage response (DDR) in LN229 and T98G cells. mRNA expression levels were determined by qRT-PCR, normalized to a matrix of 3 reference genes (18S rRNA, δ-amino-laevulinate-synthase, and β2-microglobulin), and calibrated to the results of untransformed human astrocytes. For both cell lines, three replicates were analyzed and are displayed as x-fold log2-values. **(B)** Time course analysis of DDR regulator protein expression in LN229 and T98G cells upon HSP90i by 10 nM NW457. Arrowheads indicate the bands that were used for quantification. Protein levels were normalized to a matrix comprising vinculin and α-tubulin and are depicted as x-fold log2-values compared to the 0 h controls. **(C)** Immunofluorescence microscopy of γH2AX and 53BP1 DNA damage repair foci in LN229 and T98G cells upon irradiation at 2 Gy ± HSP90i by NW457. Cells were treated with NW457 (10 nM) or DMSO for 24 h, irradiated, and fixed at the indicated times. Cells were stained for γH2AX, 53BP1, and DNA and subjected to deconvolution immunofluorescence microscopy. Scale bar depicts 10 µm. **(D)** Quantification of DNA damage repair kinetics from **(C)**. γH2AX and 53BP1 double-positive foci in at least 20 randomly picked nuclei were counted by hand. Individual data points with superimposed means ± 95% confidence intervals are displayed, and overall curve comparison was performed by two-way ANOVA. **(E)** Clonogenic survival of LN229 and T98G cells upon irradiation at 0–8 Gy ± HSP90i by NW457. Cells were pre-treated with 10 nM NW457 or DMSO for 24 h followed by irradiation at the indicated doses, and colony formation was allowed for 13 d ± continuous NW457 treatment. Individual data points of 4 independent experiments are shown, linear-quadratic regression lines are superimposed, and overall curve comparison was performed by two-way ANOVA.

A high degree of inherent radioresistance belongs to the signature hallmarks of GBM (6, 8). On the molecular level, GBM radioresistance is considered to derive from constantly activated DNA damage response (DDR) mechanisms driven by the overexpression of oncogenes, persistent replicative stress, and other so far unknown reasons (45, 46). Previous studies have shown that HSP90 plays an important role in DDR function *via* its crucial involvement in folding and stabilizing DDR proteins and/or multi-protein complexes (21, 22). Accordingly, the present study was designed to examine whether HSP90 inhibition (HSP90i) can efficiently sensitize experimental model systems of GBM to ionizing irradiation *in vitro* and *in vivo* as a multi-target approach of pharmacological interference with the DDR (26, 47, 48). For our study, we made use of two human and one mouse GBM cell line with distinct alterations in the loci of TP53, MGMT, CDKN2A, PTEN, and IDH1/2 as described for primary GBM (**Table 1**) (51, 52). Initial qRT-PCR profiling confirmed that the human GBM cell lines LN229 and T98G show a broad-range upregulation of diverse DDR regulators as compared to normal human astrocytes suggesting that DDR activity is indeed increased—irrespective of the O6-methylguanine-DNA-methyltransferase (MGMT) status (**Figure 1A** and **Supplementary Figure 1A**) (53). The highest levels of overexpression were detected for the replication and DDR-associated nucleases FEN1 and EXO1, members of the DNA double-strand break (DSB) detecting MRN complex (MRE11 and NBN), the DNA helicases BLM and PALB2, the single-strand binding protein RPA1, and members of the XRCC family which are involved in non-homologous end joining (XRCC4 and XRCC6) and alternative non-homologous end joining (XRCC1).

**Table 1:** Characteristics of the GBM cell lines used in the present study.

Cell Line	LN229	T98G	GL261
**Species**	Human	Human	Mouse
**Sex**	Female	Male	No Y chromosome detectable
**Age**	60 years	61 years	–
**MGMT status**	Promoter methylated, mRNA not detectable	Promoter not methylated, mRNA detectable	mRNA weakly detectable
**IDH1/2 status**	Wildtype	Wildtype	Wildtype
**TP53 status**	P98L mutation Function unclear	M237I mutation Dominant negative	R153P mutation Dominant negative
**CDKN2A status**	Null	Null	Null
**PTEN status**	Wildtype	L42R	Wildtype

Data were compiled from Ishii et al. (49), Cellosaurus (50), ATCC, and own unpublished data.

We then tested whether HSP90i can interfere with DDR overexpression and treated the cells with NW457, a pochoxime-derived HSP90 inhibitor with documented radiosensitizing potential and improved pharmacokinetic profile (27–30, 32). Time course westernblot analyses of diverse DDR proteins revealed different clusters of responses in LN229 and T98G cells with several common motifs (**Figure 1B**). Whereas the protein levels of few individual DDR regulators, such as NBN and RBBP8, increased upon HSP90i by NW457, clearly more candidates were downregulated, including a core cluster of CHK1, RAD51, and DNA2 with a particularly strong decrease in protein levels. Other downregulated DDR proteins comprised NHEJ1, KU80, XRCC3, ATR, CHK2, LIG4, and RPA1, and for some candidates mixed responses were observed. Our findings confirm and complement previous reports showing the critical dependence of certain DDR regulators on HSP90 chaperoning function (54–56). However, our data disclose also several DDR factors with so far unknown HSP90 dependence, including DNA2, an end resecting DNase with important functions in replication and DSB repair (57–60), and NHEJ1, a scaffold protein that binds to and assists DNA ligase 4 (LIG4) in DSB repair (61). Overall, HSP90i affected proteins of various DDR pathways – at least in the GBM cell lines used in our study – thereby providing a strong rationale for combined modality approaches of HSP90i and radiotherapy. Importantly, downregulation of DDR proteins occurred already at very low concentrations of NW457 (10 nM) which *per se* exhibited only marginal cytotoxicity even during prolonged treatment—a characteristic which is of special interest for potential future clinical translation (**Supplementary Figure 1B**). These findings confirm the notion that DDR regulators, compared to other HSP90 client proteins, are particularly sensitive towards HSP90i (22).

In order to examine the consequences of HSP90i on DDR function, LN229 and T98G cells were pre-treated with NW457 for 24 h, irradiated at 2 Gy, and subjected to immunofluorescence staining for phosphorylated histone H2AX (γH2AX) and 53BP1. The kinetics of DNA damage foci formation and clearance was quantitatively analyzed (**Figures 1C, D** **)**. HSP90i by NW457 resulted in significantly delayed clearance of γH2AX/53BP1 double-positive foci, indicating that DNA repair was obviously impaired. HSP90i also increased the overall numbers of foci as compared to the controls. This could either be due to false repair of irradiation-induced DNA damages, or irradiation-independent formation of damage sites. So far, our data show that HSP90i by low concentrations of NW457 leads to destabilization of DDR regulators in various pathways and reduced DNA damage repair capacity. In consequence, the question arises whether this also translates into reduced clonogenic survival of GBM cells upon irradiation. To this end, LN229 and T98G cells were pre-treated with NW457 for 24 h, irradiated at 0–8 Gy, and clonogenic survival was analyzed after 13 d incubation in the presence of NW457. As suggested by its marginal cytotoxicity (**Supplementary Figure 1B**), the effect of HSP90i monotherapy on clonogenic survival was modest in LN229 cells. In T98G cells it was clearly stronger, and for both cell lines this was statistically significant (**Supplementary Figure 1C**). Importantly, HSP90i by NW457 significantly reduced the clonogenic survival upon irradiation in both cell lines (**Figure 1E**), confirming our hypothesis that multi-target interference with DDR function by HSP90i at *per se* non-toxic doses suffices to sensitize resistant GBM cells to irradiation. Similar findings were very recently reported for other cancer entities (62, 63). Morphologically, the mode of cell death underlying reduced clonogenic survival upon HSP90i plus radiation was a highly disruptive, necrotic one which occurred after several rounds of aberrant mitosis and intermediate states of highly aneuploid cells with multiple and/or giant nuclei (**Supplementary Movie File 1**) (64).

### HSP90 Inhibition by NW457 Stimulates a Compensatory Transcriptional Upregulation of Genes Involved in Protein Production Accompanied by Downregulation of Genes Engaged in Survival Signaling, Cell Stemness, and Integrin Signaling

Our results suggest—at first sight—that sensitization to radiotherapy upon HSP90i by NW457 derives from the downregulation of crucial DDR mediators on the protein level. Certainly, the immediate consequences of HSP90i affect the post-translational level where abortive chaperoning leads to proteasomal degradation of HSP90 client proteins (65). Nevertheless, it is feasible to assume that this broad-range protein catabolytic remodeling can also stimulate complex responses on the transcriptome level which may contribute to radiosensitization as well. In order to address this question, we performed RNA sequencing analyses upon HSP90i by NW457 (10 nM) in LN229 and T98G cells. Differential gene expression analysis revealed an overt transcriptomic response in LN229 cells, and an attenuated but still detectable response in T98G cells (**Supplementary Figure 2A**). The overlap in up- or downregulated genes was rather small (**Supplementary Figure 2B**), possibly pointing towards a regulatory involvement of p53 (T98G have dominant negative p53^M237I^, LN229 carry p53^P98L^ with unclear functionality but intact DNA binding domain, see **Table 1** and **Supplementary Table 2**). Construction of a functional interaction (FI) network of the upregulated intersect genes revealed a clear activation of the heat shock response, comprising many chaperones and members of the heat shock protein family (**Supplementary Figure 2C**). This FI network appeared to be predominantly controlled by heat shock factor 1 (HSF1) as suggested by iRegulon analysis (**Supplementary Figure 2D**). For the common downregulated genes shared by LN229 and T98G cells, a smaller FI network was constructed, basically comprising elements of DNA repair, mitosis regulation, NOTCH signaling, and protein folding (**Supplementary Figure 2E**). iRegulon analysis suggested a rather heterogenous pattern of transcriptional regulators, including EP300, JUND, BCL3, and others (**Supplementary Figure 2F**).

Further geneset enrichment analysis (GSEA) of the transcriptomic alterations in LN229 cells upon HSP90i revealed positive enrichment of distinct MSigDB hallmark genesets comprising targets of MYC and E2F, as well as regulators of the G2/M cell cycle checkpoint (**Supplementary Figure 3A**). The FI network of the compiled leading edge genes allowed the conclusion that this was in principle a compensatory response to HSP90i treatment, because interaction clusters representing basic functions of RNA polymerase II transcription, mRNA processing and splicing, RNA transport, translation initiation, protein folding, rRNA processing, and ribosome biogenesis were identified. Moreover, clusters involved in cell cycle regulation (G2/M transition, G1/S transition, and mitosis) and DNA repair were observed among the leading edge genes. Without prior GSEA, the FI network of all significantly upregulated genes showed additional interaction clusters of transcription factor activation (AP1, GR, and p53), focal adhesion and organization of the extracellular matrix (ECM), the heat shock response, and signaling by small GTPases (RAS, RAP1, and RHO) (**Supplementary Table 2**).

In terms of transcriptional downregulation upon HSP90i, no significantly enriched MSigDB hallmark genesets (FDR < 0.1) were observed in LN229 cells. However, on the level of significantly downregulated individual genes FI network construction revealed several interaction clusters whose decreased expression may contribute to radiosensitization by HPS90i. As such, clusters involved in survival signaling (EGFR and IGF1 signaling, PI3K/AKT/mTOR signaling) and maintenance of cell stemness (NOTCH signaling) represent potential candidates, as well as clusters orchestrating integrin signaling, ECM receptor interaction, and ECM organization (**Supplementary Table 3**).

### HSP90 Inhibition by NW457 Improves the Efficacy of Fractionated Radiotherapy in an Orthotopic, Syngeneic GBM Mouse Model

**Figure 2:**
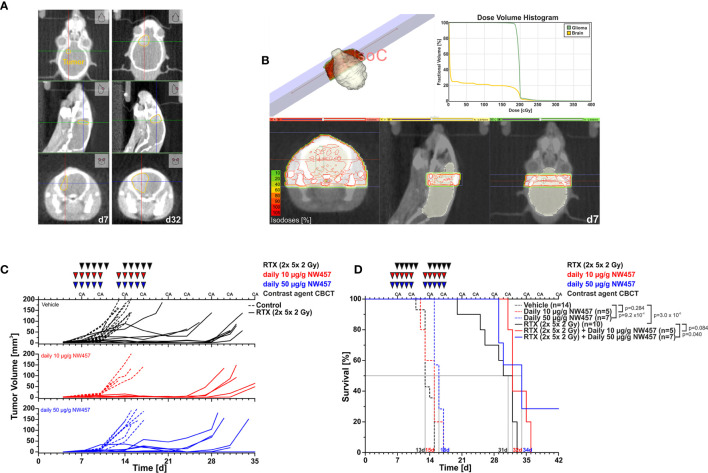
HSP90i by NW457 improves the efficacy of fractionated radiotherapy in an orthotopic, syngeneic GBM mouse model. *In vivo* performance of NW457-mediated HSP90i in combination with fractionated radiotherapy in orthotopically transplanted GL261 tumors. **(A)** Tumor localization and growth monitoring of orthotopically implanted GL261 cells in C57BL/6 mice were performed by contrast-enhanced CBCT scans and manual contouring. **(B)** Treatment plan and dose volume histogram. Two contralateral beams with 3× 9 mm^2^ collimation were used to administer 2× 5× 2 Gy. **(C)** Treatment schedule and tumor growth curves. Six days after orthotopic transplantation, mice were randomized into 6 groups (vehicle, 10 μg/g NW457, 50 μg/g NW457, 2× 5× 2 Gy + vehicle, 2× 5× 2 Gy + 10 μg/g NW457, 2× 5× 2 Gy + 50 μg/g NW457), and treatment was administered according to the indicated schedule. **(D)** Kaplan-Meier survival analysis of all treatment groups. Tumor-specific death was scored when mice showed pre-defined symptoms. p-values were obtained by log-rank test.

In the next step, we examined the performance of HSP90i plus radiotherapy *in vivo*. We made use of a syngeneic orthotopic mouse GBM transplantation model with *i.p.* injection of an *in vivo* NW457 formulation and contrast-enhanced, conebeam (CB)CT-based, fractionated radiotherapy (43) (**Figure 2**). Importantly, we first recapitulated the basic *in vitro* experiments with GL261 cells, the murine cell line that was used for transplantation. This cell line showed a particularly strong downregulation of DDR proteins upon HSP90i (**Supplementary Figures 4A, B** **)** and did not tolerate permanent NW457 incubation in colony formation assays so that NW457 needed to be removed after the 24 h pre-incubation time (**Supplementary Figures 4C, D** **)**. Nevertheless, this treatment was already sufficient to facilitate radiosensitization of GL261 cells which have been described to exhibit particularly high levels of intrinsic treatment resistance (66).

Upon transplantation of GL261 cells into the right hemispheres of C57BL/6 mice, tumor progression was monitored by contrast-enhanced CBCT scans over time (**Figure 2A**). Starting at d7 after implantation, mice were subjected to CBCT-guided, fractionated radiotherapy with 2× 5× 2 Gy using two contralateral beams at 3× 9 mm^2^ collimation (**Figure 2B**), and NW457 (or the vehicle control) was administered 24 h before each radiation treatment (10 or 50 µg/g *i.p.*). Tumor growth follow-up was accomplished by serial contrast-enhanced CBCT scans, and animals were sacrificed when reaching the pre-defined humane endpoints. Of note, d7 CBCT scans confirmed that tumor volumes were statistically not significantly different across different treatment groups at the start of therapy (**Supplementary Figure 5A**).

Overall, the treatment was tolerated well, and no significant differences in body weight in response to the treatment were observed (**Supplementary Figure 5B**). Tumors of vehicle-treated animals grew exponentially, and monotherapy with HSP90i delayed tumor growth only marginally (**Figure 2C**). Fractionated radiotherapy exerted strong inhibitory effects on tumor growth, but responses were rather heterogeneous among the animals in this group. Additional HSP90i delayed tumor growth even further—yet compared to the effects observed *in vitro*, the *in vivo* performance was not as strong as expected. These findings basically mirrored the Kaplan-Meier survival analyses (**Figure 2D**). HSP90i in mono-agent settings had only minor impact on animal survival, although this reached statistical significance compared to the vehicle controls at 50 µg/g NW457. Upon radiotherapy alone, animals revealed clearly prolonged survival, albeit again with heterogenous responses. Survival times were further increased by additional HSP90i, although to a rather limited, yet statistically significant extent in case of 50 µg/g NW457. Interestingly, two animals of the combined modality group (radiotherapy + 50 µ/g NW457) showed full tumor remission translating into persistent survival until the end of the experiment (**Supplementary Figure 5C**). In summary, these data indicate that HSP90i by NW457 can complement and improve the efficacy of fractionated radiotherapy in the used GBM *in vivo* model. Nevertheless, since the effects observed *in vitro* clearly outcompete the performance *in vivo*, further optimization of HSP90 inhibitor substances and/or treatment sequences would be needed.

### HSP90i by NW457 Attenuates Irradiation-Induced Hypermigration and Invasiveness of GBM Cells *In Vitro* and *In Vivo*

**Figure 3:**
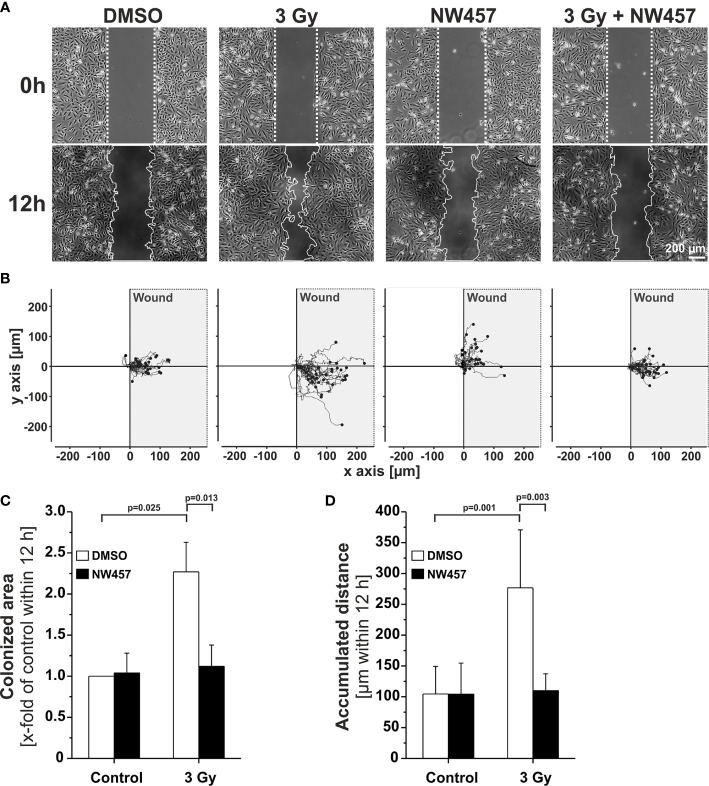
HSP90i by NW457 counteracts GBM cell hypermigration in response to irradiation *in vitro*. **(A)** Live-cell imaging of the migratory behavior of GBM cells in wound healing assays. LN229 cells were seeded into Ibidi µ-slides with silicon “wound” inserts, pre-treated with 30 nM NW457 or DMSO for 24 h, irradiated at 3 Gy where indicated, and analyzed by live-cell microscopy for 12 h. “Wound” edges at the beginning and the end of the experiment are delineated by white dotted lines, and scale bar depicts 200 µm. **(B)** Trajectory plots showing the migratory paths of at least 25 randomly picked and manually tracked cells from **(A)**. **(C)** Relative quantification of the colonized area (depicted as x-fold values of controls) is shown as means ± s.d. of 3 independent experiments. Group comparisons were performed by Student’s *t*-tests. **(D)** Relative quantification of accumulated distances per cell. Means ± s.d. of at least 25 randomly picked cells are shown. Group comparisons were performed by Student’s *t*-tests.

**Figure 4:**
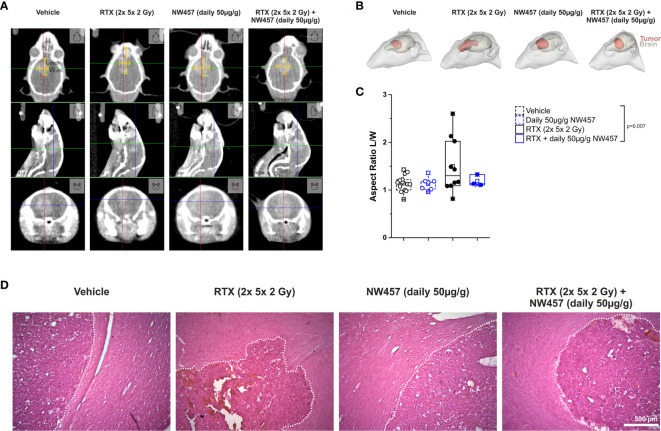
HSP90i by NW457 attenuates GBM invasiveness in response to irradiation *in vivo*. Analysis of tumor morphology upon treatment at the time of animal sacrifice. **(A)** Contrast-enhanced CBCT scans of mice from different treatment groups. Tumor dimensions (width, length) are depicted by yellow bars in the coronal section (upper panels). **(B)** 3D reconstruction of tumors (red) and brains (grey) in mice from each treatment group as generated in 3D-Slicer. **(C)** Quantitative aspect ratio analysis of tumor length/width from each treatment group. p-value was calculated by one-way ANOVA (RTX vs. all other groups). **(D)** Hematoxylin/eosin (HE) stainings of tumors from all treatment groups. Tumor borders are highlighted by a dotted white line, and scale bar depicts 500 µm.

Several previous studies have shown that non-lethal irradiation results in accelerated GBM cell migration—a phenomenon with implications for relapse and treatment failure (67, 68). We therefore examined, whether HSP90i by low-dose NW457 treatment does interfere with GBM cell migration. LN229 cells were treated with NW457 for 24 h, irradiated at 3 Gy, and their migratory behavior was analyzed in wound healing setups by live-cell imaging (**Figure 3A** and **Supplementary Movies File 2**). Migration was quantitated by the colonized area and the accumulated distance per cell over time as determined by tracking of at least 25 randomly picked cells per condition (**Figures 3B–D**). With both approaches, the basal migratory activity of LN229 cells was observed to be significantly increased by radiation at 3 Gy, and this was almost completely reversed by pre-treatment with NW457. Thus, HSP90i, in addition to its radiosensitizing potential, does also efficiently counteract irradiation-induced hypermigration of GBM cells. In order to test whether this also holds true *in vivo*, we characterized the morphology of the tumors from our *in vivo* experiments. The aspect ratios of L axes (cranial-caudal, 90° to beam axes) and W axes (left-right, 0° to beam axes) were determined in contrast-enhanced CBCT scans of all mice at the day of sacrifice, and exemplary 3D reconstructions were generated (**Figures 4A–C**). Tumors of mice from the radiotherapy-only group showed significantly distorted aspect ratios and caudal-cranially stretched 3D reconstructions, implying tumor progress orthogonally to the irradiation field. This effect was fully reversed by additional NW457 treatment. Histologically, tumors from irradiated mice revealed clearly more invasive borders than tumors from vehicle or NW457-only-treated mice, and also more and larger areas of hemorrhage (**Figure 4D**). Intriguingly, this morphotype was also fully reversed upon co-treatment with NW457, indicating that HSP90i does not only improve the therapeutic efficacy of radiotherapy but also counteracts GBM cell migration and tumor invasiveness in response to radiotherapy. These findings clearly strengthen the attractiveness of HSP90i as a partner for radiotherapy in combined modality settings.

## Discussion

Radiotherapy is a fundamental part of the standard of care for the treatment of glioblastoma (GBM) (69). However, treatment failure and (in-field) recurrence are frequent and form the basis for the dismal prognosis of this devastating disease (1). Significant advances in radiotherapy treatment and image-guidance technology as well as the addition of temozolomide (TMZ), a DNA alkylating chemotherapeutic drug, have led to modestly improved outcomes (2, 3), yet continued development remains urgently needed. GBM is characterized by a high level of inherent radioresistance which is considered to derive from overexpression of DNA damage response (DDR) genes and basally increased DDR activity (6, 8, 9, 45, 46). In this context, the molecular chaperone HSP90 is of particular interest and represents a promising target for radiosensitization approaches, since several key regulators of the DDR are known to crucially depend on HSP90 folding assistance (70–75). However, in single-agent settings administration of HSP90 inhibitors was frequently associated with side effects of relevant severity, including gastrointestinal toxicity and hepatoxicity, because the concentrations needed to achieve anti-tumor effects—despite the relative selectivity for cancer versus normal cells—were rather high, and the employed substances exhibited suboptimal toxicity profiles and poor pharmacokinetic features (76). Intriguingly, quantitative mass spectrometric analyses revealed that pathways of the DDR are among the most sensitive ones in cancer cells that are perturbed by HSP90i already at very low inhibitor concentrations (22). Furthermore, a recent study showed that administration of very low, non-toxic doses of an HSP90 inhibitor of the third generation results in DDR protein disintegration in HNSCC and pancreatic cancer cells, while this was not observed in non-transformed, normal cells (62). This would allow targeting DNA damage repair mechanisms in cancer cells while not affecting the normal tissue and—in combination with radiotherapy—would imply a kind of biologically driven increase in radiation dose selectively at the tumor. Given that treatment-associated radionecrosis represents a major dose limiting factor in GBM radiotherapy, targeted radiosensitization of the tumor by HSP90i appears specifically attractive for this cancer entity, ideally in combination with modern high precision, image-guided radiotherapy (69).

In the present study, we report that treatment with very low concentrations of the pochoxime-derived HSP90 inhibitor NW457 which *per se* exhibit only limited cytotoxicity leads to DDR protein disintegration in GBM cells. We observed several key regulators of the DDR to be affected by HSP90i, previously published HSP90 client proteins as well as DDR regulators with so far unknown HSP90 dependence (32, 54–56, 62). The cluster with the strongest decrease in protein levels upon HSP90i treatment in human and mouse GBM cell lines comprised CHK1, RAD51, DNA2, and NHEJ1. Accordingly, HSP90i represents a multi-target approach and affects various DDR pathways, including upstream checkpoint signaling (CHK1), double-strand break (DSB) repair by homologous recombination (RAD51), and non-homologous end-joining (NHEJ1), as well as crosslink repair and DNA replication (DNA2). This may explain why HSP90i is such a potent means of radiosensitization compared to mono-target approaches, for instance PARP inhibition (26, 77). Nevertheless, due to the correlative nature of our observations we cannot exclude that other mechanisms, such as the HSP90i-triggered proteasomal degradation of non-DDR proteins or the observed transcriptional downregulation of various genes, for instance genes involved in survival and/or integrin signaling, contribute to radiosensitization (78–81).

Functionally, degradation of DDR regulators upon HSP90i was accompanied by delayed DNA damage repair kinetics and significantly impaired clonogenic survival upon irradiation. *In vivo*, HSP90i by NW457 augmented the efficacy of fractionated radiotherapy in an orthotopic, syngeneic GBM mouse model, both in terms of tumor progression and survival. However, the observed effects were not as strong as expected from the convincing *in vitro* results. This may be due to limited GBM penetration by the inhibitor *in vivo*, although the family of pochoxime-derived HSP90 inhibitors has been shown to exhibit favorable brain pharmacokinetic profiles (82). Nevertheless, a very recent study with an orthotopic patient-derived GBM model reported similar therapeutic efficacy of a related pochoxime-derived HSP90 inhibitor in combination with whole brain irradiation (83). So, inhibitor substances with improved brain pharmacokinetic profiles, optimized formulations and/or administration routes, and/or fine-tuned treatment sequences may help to fully develop the synergistic potential of HSP90i and radiotherapy for the treatment of GBM.

In addition to its radiosensitizing effects, we observed that HSP90i by NW457 did reverse irradiation-induced GBM cell hypermigration *in vitro* and GBM invasiveness *in vivo*. This is of relevant interest, since GBM cells which survive radiotherapy and evade the target volume of radiotherapy may drive tumor relapse and dissemination. Our findings are in line with other reports showing that HSP90i efficiently decreases migration and invasion of human GBM cell lines (84–86). Although the detailed mechanisms of action remain elusive, we assume that the downregulation of migration regulating proteins is of importance in this scenario. On the protein level, mediators of protein (tyrosine) kinases have been reported to be particularly sensitive to HSP90i, and these are crucial regulators of migration-relevant signaling cascades (22). Additionally, our study shows that HSP90i stimulated the transcriptional downregulation of several interaction clusters involved in migratory processes, including integrin signaling, ECM receptor interaction, and signaling by small and large GTPases. HSP90i may thus offer a means to interfere with the highly infiltrative GBM phenotype which worsens with radiotherapy and represents another hallmark of this cancer entity contributing to its poor prognosis.

It should be noted, that HSP90i has also been shown to synergize with TMZ treatment in orthotopic models of GBM (87). This raises the question whether a triple combination of HSP90i and the current clinical standard of TMZ-based radiochemotherapy may improve the therapeutic outcome even further.

In conclusion, our study shows that HSP90i by low doses of NW457 potently interferes with the DDR in GBM cells leading to significant sensitization towards radiotherapy *in vitro*. The *in vivo* performance of this combined modality approach was less convincing than expected, although tumor growth was clearly delayed, survival was significantly prolonged, and radiation-induced invasive tumor morphology was reverted. Hence, our data reveal that the combination of HSP90i and radiotherapy is a promising strategy for GBM treatment whose performance needs to be further optimized by improved inhibitor substances, better formulations and/or administration routes, and fine-tuned treatment sequences.

## Data Availability Statement

The RNA sequencing datasets presented in this study can be found in online repositories. The names of the repository/repositories and accession number(s) can be found here: NCBI GEO: GSE164717. All other datasets are available from the corresponding author upon reasonable request.

## Ethics Statement

The animal study was reviewed and approved by the *Regierung von Oberbayern*.

## Author Contributions

KL, MN, CB, AAF, HZ, KU, AKW and MO conceived and designed the experiments. MO, VA, KS, LKi, BS, AN, JM, LKr, NS, JH, KU and KL performed the experiments and analyzed the data. NW provided the HSP90i NW457. MO and KL wrote the manuscript with support from KU and JH. All authors discussed the results, commented on and revised the manuscript.

## Funding

This work was in part funded by the *Bildungsministerium fuer Bildung und Forschung* [02NUK047C and the German Cancer Consortium (DKTK)], the *Deutsche Forschungsgemeinschaft* (INST 409/126-1 FUGG, INST 409/20-1 FUGG, and INST 409/22-1 FUGG), and the FoeFoLe Program of the Medical Faculty of the LMU Munich.

## Conflict of Interest

All commercial rights on pochoximes (including *epi*-pochoxime F) were licensed by Nexgenic Pharmaceuticals (New York, NY, USA). NW consulted Nexgenic Pharmaceuticals, and received funding from Nexgenic Pharmaceuticals.

The remaining authors declare that the research was conducted in the absence of any commercial or financial relationships that could be construed as a potential conflict of interest.

## Glossary

53BP1: p53-binding protein 1
ATM: ataxia telangiectasia mutated
ATR: ataxia telangiectasia and Rad3 related
BLM: Bloom syndrome helicase
BRCA1: breast cancer gene 1
BRCA2: breast cancer gene 2
BRIP1: BRCA1-interacting protein 1
CBCT: conebeam computed tomography
CDKN1A: cyclin dependent kinase inhibitor 1A
CHK1: checkpoint kinase 1 (gene symbol CHEK1)
CHK2: checkpoint kinase 2 (gene symbol CHEK2)
DCLR1C: DNA cross-link repair protein 1C
DNA2: DNA replication ATP-dependent nuclease 2
DNA-PKcs: catalytic subunit of DNA-dependent protein kinase (gene symbol PRKDC)
DDR: DNA damage response
DMSO: dimethyl sulfoxide
DSB: double-strand break
EORTC: European Organization for Research and Treatment of Cancer
EXO1: exonuclease 1
FANCA: Fanconi anemia complementation group A
FEN1: Flap endonuclease 1
FCS: fetal calf serum
GBM: glioblastoma
γH2AX: phosphorylated histone variant 2AX (pS139)
HE: hematoxylin and eosin
HSP70: heat shock protein 70
HSP90: heat shock protein 90
HSP90i: heat shock protein 90 inhibition
KU70: ATP-dependent DNA helicase subunit KU70 (gene symbol XRCC6)
KU80: ATP-dependent DNA helicase subunit KU80 (gene symbol XRCC5)
LIG4: DNA-ligase 4
MGMT: O6-methylguanine-DNA-methyltransferase
MRE11: meiotic recombination protein 11
MRN: MRE11-RAD50-NBN
NBN: Nibrin
NCIC: national cancer information center
NHEJ1: non-homologous end joining protein 1
p21: cyclin-dependent kinase inhibitor p21 (gene symbol CDKN1A)
p53: tumor suppressing protein p53 (gene symbol TP53)
PALB2: partner and localizer of BRCA2
PARP1: poly(ADP-ribose)-polymerase 1
PARP2: poly(ADP-ribose)-polymerase 2
PS: pencillin/streptomycin
RAD50: DNA repair protein RAD50
RAD51: DNA repair protein RAD51
RAD52: DNA repair protein RAD52
RAD54: DNA repair protein RAD54
RBBP8: Retinoblastoma-binding protein 8
RPA1: replication protein A1
qRT-PCR: quantitative realtime reverse transcription polymerase chain reaction
RTX: radiotherapy
STR: short tandem repeat
TMZ: temozolomide
XRCC1-5: x-ray repair cross-complementing protein 1-5
