# Supplementary material for: Inhibition of HSP90 as a Strategy to Radiosensitize Glioblastoma: Targeting the DNA Damage Response and Beyond
Source: Front Oncol. 2021 Mar 17;11:612354. doi: 10.3389/fonc.2021.612354 (PMC8011354; doi:10.3389/fonc.2021.612354)
Supplement: Supplementary file 1 [file DataSheet_1.zip › Data Sheet 1.pdf]

Inhibition of HSP90 as a strategy to radiosensitize glioblastoma: Targeting the DNA damage response and beyond

Michael Orth[1], Valerie Albrecht[1], Karin Seidl[1], Linda Kinzel[1], Kristian Unger[2], Julia Hess[2], Lisa Kreutzer[2], Na Sun[3], Benjamin Stegen[1,4,5], Alexander Nieto[1], Jessica Maas[1], Nicolas Winssinger[6], Anna A. Friedl[1], Axel K. Walch[3], Claus Belka[1,4,7], Horst Zitzelsberger[2,7], Maximilian Niyazi[1], and Kirsten Lauber[1,4,7,*]

[1] Department of Radiation Oncology, University Hospital, LMU Munich, Munich, Germany
[2] Research Unit Radiation Cytogenetics, Helmholtz Center Munich, German Research Center for Environmental Health GmbH, Neuherberg, Germany
[3] Research Unit Analytical Pathology, Helmholtz Center Munich, German Research Center for Environmental Health GmbH, Neuherberg, Germany
[4] German Cancer Consortium, Munich, Germany
[5] German Cancer Research Center, Heidelberg, Germany
[6] Department of Organic Chemistry, NCCR Chemical Biology, University of Geneva, Geneva, Switzerland
[7] Clinical Cooperation Group Personalized Radiotherapy in Head and Neck Cancer, Helmholtz Center Munich, Neuherberg, Germany

*** Correspondence:**
Kirsten Lauber, University Hospital, LMU Munich, Department of Radiation Oncology, 81377 Munich (Germany), Phone: +49 89 440076740, Fax: +49 89 440076770
Email: kirsten.lauber@med.uni-muenchen.de

Running Title: HSP90i-mediated radiosensitization of glioblastoma

Keywords: HSP90 inhibition, HSP90i, NW457, radiosensitization, glioblastoma, radiotherapy, hypermigration

Manuscript information:

Total Word Count: 6114 (excluding Abstract, References, and Figure legends)
Abstract Word Count: 288
References: 87
Figures: 4
Tables: 1
Supplemental Figures: 5
Supplemental Movie Files: 2
Supplemental Tables: 3

Supplement

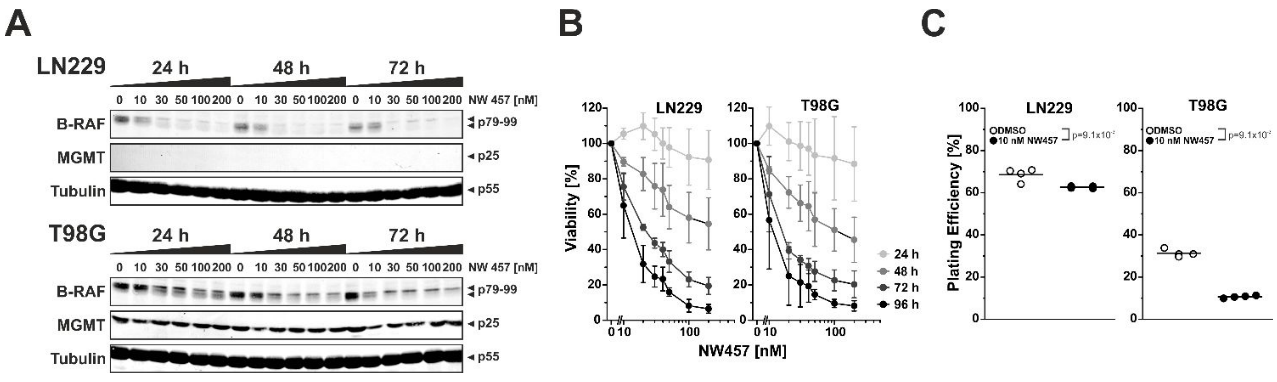

Supplemental Figure 1: NW457 is an HSP90 inhibitor with little toxicity for GBM cells in the low-dose range. (A) Protein expression levels of B-RAF and MGMT in LN229 and T98G cells upon treatment with 0-200 nM NW457 for 24-72 h. Bands are depicted by arrowheads and molecular weights (in kD). α-tubulin served as loading control. (B) Viability of LN229 and T98G cells after treatment with 0-200 nM NW457 for 24-96 h as determined by Alamar Blue assays. Means ± s.d. of 4 independent experiments are shown. (C) Clonogenic survival of LN229 and T98G cells after treatment with 10 nM NW457 without additional irradiation as determined by colony formation assays (see Fig. 1). Data points of 4 independent experiments are shown with means superimposed. p-values were calculated by one-way ANOVA.

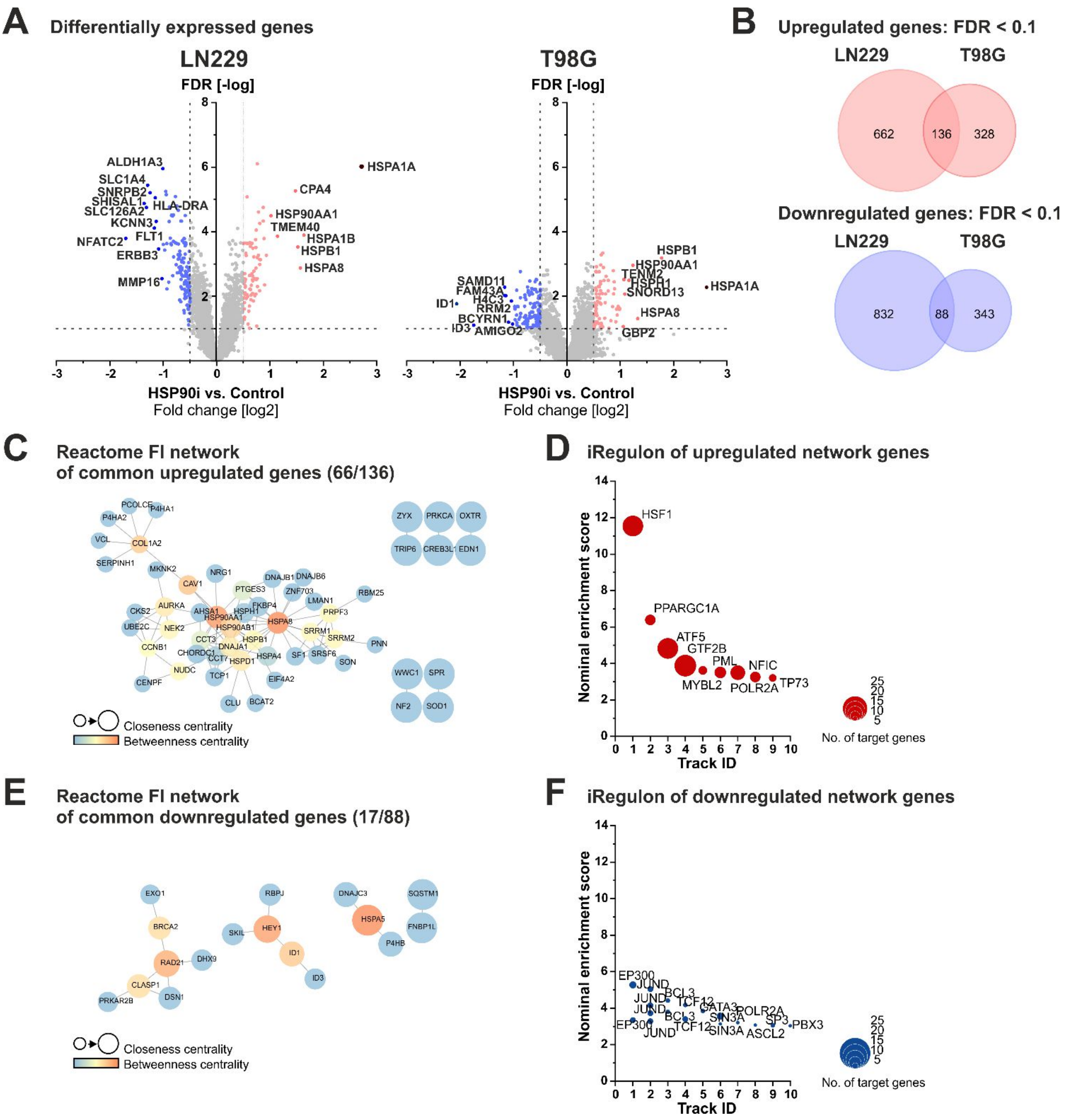

Supplemental Figure 2: Common transcriptional alterations in LN229 cells and T98G cells upon NW457-mediated HSP90i reveal transcriptional upregulation of the heat shock response.

LN229 cells and T98G cells were treated with NW457 (10 nM) for 24 h in 5-6 biological replicates per condition, and extracted RNA was subjected to RNA sequencing with downstream data analysis. (A) Volcano plots depicting differentially expressed genes. Genes with up- or downregulation of ≥ |0.5| fold change [log2] and FDR < 0.1 are colored, others are displayed in grey. (B) Venn diagram of significantly up- and downregulated genes in LN229 and T98G cells. (C) Reactome FI network of common upregulated genes from (B). Only connected genes are displayed. (D) iRegulon analysis of upregulated network genes from (C) showing the nominal enrichment score in dependence of the track ID. (E) Reactome FI network of common downregulated

genes from (B). Only connected genes are displayed. (F) iRegulon analysis of downregulated network genes from (E).

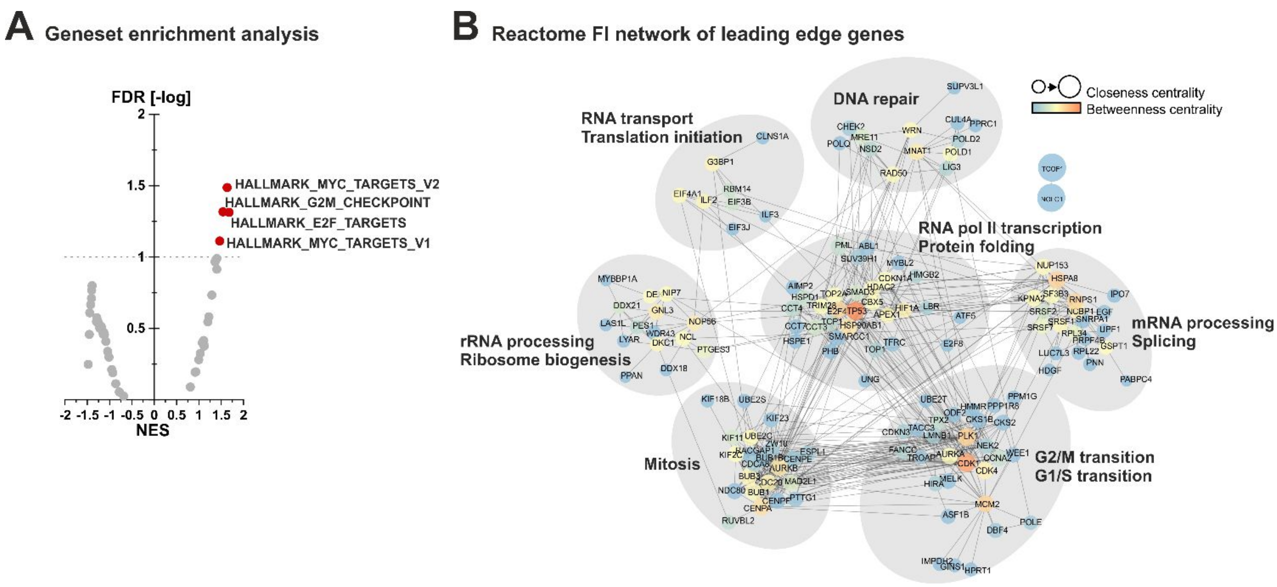

Supplemental Figure 3: Geneset enrichment analysis (GSEA) upon NW457-mediated HSP90i in LN229 cells discloses a compensatory transcriptional upregulation of genes involved in protein production, cell cycle regulation, and DNA repair.

Log2 expression values were subjected to geneset enrichment analysis (GSEA) utilizing the MSigDB Hallmark geneset collection. (A) Volcano plot showing nominal enrichment scores and FDR [-log] values. Significantly enriched genesets (FDR < 0.1) are colored and labeled, others are displayed in grey. (B) Reactome FI network of all leading edge genes from the significantly enriched genesets shown in (A). Only connected genes are displayed. Clustered gene modules were subjected to pathway enrichment analysis.

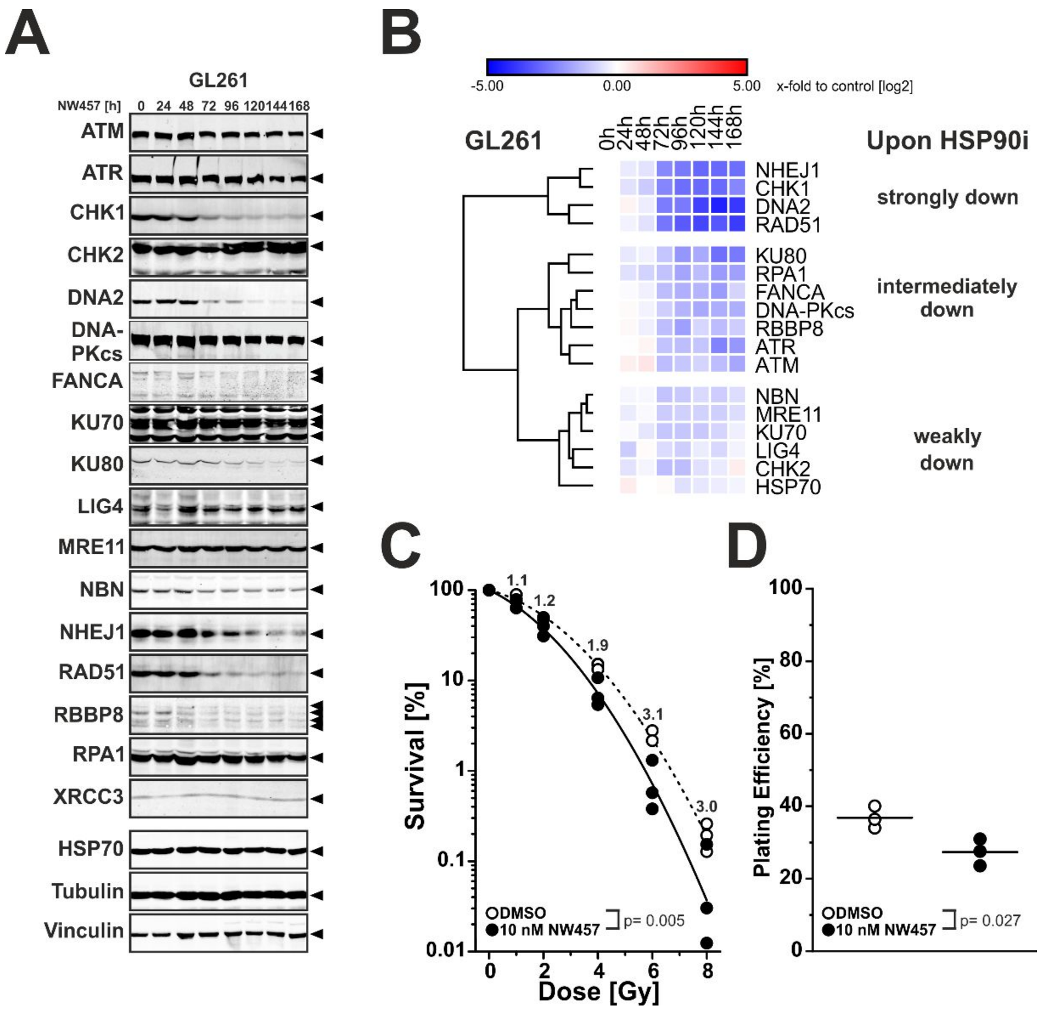

Supplemental Figure 4: NW457-mediated HSP90i results in radiosensitization of GL261 cells *in vitro*.
(A) Time course analysis of DDR regulator protein expression in GL261 cells as in Fig. 1B. Bands marked by arrowheads were used for quantification. (B) Heat map showing relative expression levels of DDR regulator proteins in GL261 cells upon treatment with 10 nM NW457. Protein levels were normalized to a matrix comprising vinculin and α-tubulin and are depicted as x-fold log2-values compared to the 0 h controls. (C) Clonogenic survival of GL261 cells upon irradiation at 0-8 Gy ± HSP90i by NW457. Cell were pre-treated with 10 nM NW457 or DMSO for 24 h followed by irradiation at the indicated doses, and colony formation was allowed for 13 d without further NW457 treatment. Individual data points of 3 independent experiments are shown, and overall curve comparison was performed by two-way ANOVA. (D) Clonogenic survival of GL261 cells after treatment with 10 nM NW457 without additional irradiation as determined in (C). Data points of 3 independent experiments are shown with means superimposed. p-value was calculated by one-way ANOVA.

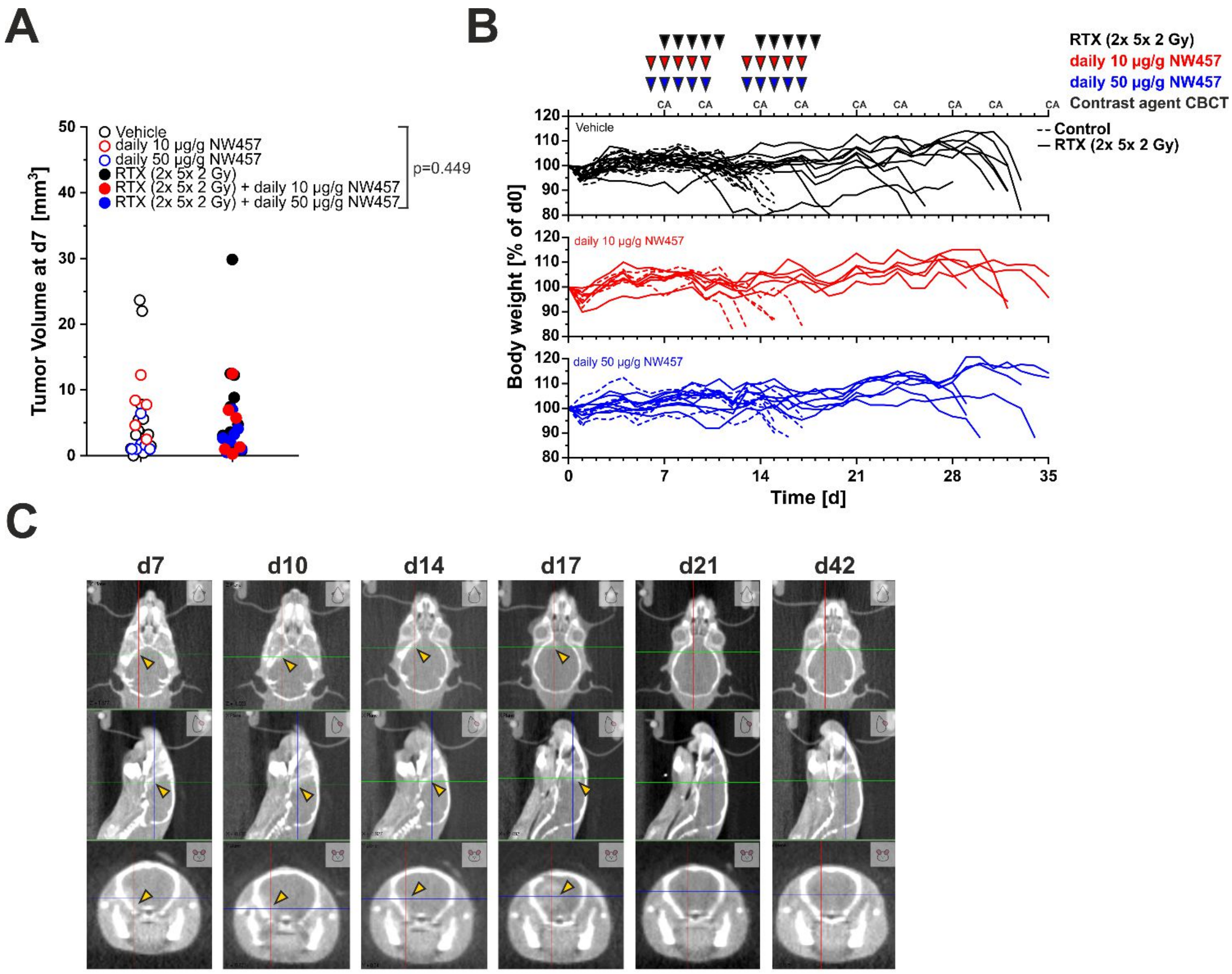

Supplemental Figure 5: Tumor volumes at the start of radiotherapy, body weight curves of animals, and exemplary series of contrast-enhanced CBCT scans of a survivor animal.

(A) Tumor volumes of all animals at the start of radiotherapy on d7. Group comparison was performed by one-way ANOVA. (B) Body weight curves of all animals along the experiment as % of body weight on d0. (C) Exemplary series of contrast-enhanced CBCT scans of a survivor animal. Tumor is depicted by yellow arrowheads.

Supplemental movies 1.pptx

Supplemental movies file 1: Time-lapse microscopy upon irradiation ± NW457-mediated HSP90i in LN229, T98G, and GL261 cells.

Cells seeded into Ibidi µ-slides were treated with 10 nM NW457 or DMSO for 24 h followed by irradiation at 4 Gy. Live-cell imaging was performed over 12 d in 12 min intervals. Scale bars represent 100 µm.

Supplemental movies 2.pptx

Supplemental movies file 2: Time-lapse microscopy upon irradiation ± NW457-mediated HSP90i in LN229 cells.

Cells seeded into Ibidi µ-slides were treated with 30 nM NW457 or DMSO for 24 h followed by irradiation at 3 Gy. Live-cell imaging was performed over 12 h in 3 min intervals. Scale bars represent 200 µm.

Supplemental Table 1: Sequences of primer pairs used for Realtime PCR analyses.

Target gene	Forward primer	Reverse primer
**18S rRNA**	CGGCTACCACATCCAAGGAA	AGCTGGAATTACCGCGGC
**B2M**	TGCTCGCGCTACTCTCTCTTTC	TCTCTGCTGGATGACGTGAGTAAAC
**ALAS**	TCCACTGCAGCAGTACACTACCA	ACGGAAGCTGTGTGCCATCT
**ATM**	CTGGAAGAAGCACAAGTATTCTGGG	TGGGATTGTTCGCTGCACA
**ATR**	GGTCACCACCAGACAGCCTAC	GAACATCACCCTTGGACCAGA
**BLM**	ATGGACATCGCCAGCTGAAG	GCAAAGTAGTGGGAAGATACGGG
**BRCA1**	GCTATTTCTGGGTGACCCAGTC	GATTCTCTTGCTCGCTTTGGA
**BRCA2**	GTTTGTGAAGGGTCGTCAGACA	AACTAAGGGTGGGTGGTGTAGCT
**BRIP1**	GTGTGCTGTAGCCTCATTAATTGG	GTTAAGAGCATTTCATTTCCACTCC
**CHEK1**	ATGCCTGAACCAGATGCTCAG	GAGGTTATCCCTTTCATCCAACAG
**CDKN1A**	CTGGAGACTCTCAGGGTCGAAA	AGTGGTAGAAATCTGTCATGCTGGT
**CHEK2**	CTCTTGGAAGTGGTGCCTGTG	GGGTCTGCCTCTCTTGCTGA
**DCLRE1C**	CGGAGCCAAAGTATAAACCACTG	TCATCTTCCTCCTCTGAGTCT
**DNA2**	TTCCTTAATACAGACAAGGTTCCAGC	CATCCAGCCTTAACAAAAATGGAG
**PRKDC**	AACAACTTTATGGTGGCCATGG	ACTGGCAGAAACTGTGTAGCGG
**EXO1**	AGCTACGCTGGGCAATATGTTG	AATGGGCAGGCATAGCAGTG
**FANCA**	GACTGTGGAGCAGAGAAAGAAGCT	CATACTGTGTGCCAATAAATACTGAGC
**FEN1**	GTGTCCCAAAGGCCAGTCATC	GGGCCACATCAGCAATTAGTTT
**XRCC6**	GGGCTCCTTGGTGGATGAGT	GCTTCCAGAACCTTCATTATCGTG
**XRCC5**	GAGCATAGACTGCATCCGAGC	CTGAAAACTTAATGGCTTCTTCCC
**LIG4**	AAGCCTTGGAGCTTCGGTTT	TCTGCAACACGACTATGATCTTCC
**MRE11**	TGGAGAAGATTGAAGAAATGCTTGA	CCACTATAGTCCACTCGCAGTCG
**MGMT**	GCAATCCTGTCCCCATCCT	AGACCACTCTGTGGCACGG
**NBN**	CAGGAAAACTTCCACACATCATTG	TTTGTACCTCCATTTCCTGCCTTA
**PALB2**	TGTATAATCTCGTGTGTGTAGCTTTGG	CAATGCCCTGATCTCTCTGATTTC
**PARP1**	GCCCGTGACAGGCTACATGT	TTGGGTCTCCCTGAGACGTATG
**PARP2**	TGGGTGGGAATCTTGAGCC	CTTTCCCAAACATGTAACCTGTGA
**RAD50**	CGAAGTACCTATCGTGGACAAGATATT	TCGGCATCAGACCGTATTTCTAT
**RAD51**	GCAGTGGCTGAGAGGTATGGTC	TGGTGGTCTGTGTTGAACGC
**RAD52**	CAGCAGAAGAGTACCAGGCCAT	TCAATGTAGCACACCTTCTGGC
**RAD54**	GAGTATGAAGCAGAGAAGAAGGGACT	CCAGTTGGTATGTTGAAACGCAT
**RBBP8**	CTGTAATAGATACAAAGGATGGCAGTCA	AACCAATGTACAGTCCATGTCCACT
**RPA1**	AGGCGACAAGCCGGACTAC	AGGCTTGGTACATGCAGTTCTCTT
**NHEJ1**	AGCTAGTCCTTCCCTGGTCTCC	CCCATCAGAGGACGAATCAAAT
**TP53**	CCGCCTGAGGTTGGCTC	CGCCCATGCAGGAACTGT
**XRCC1**	GAGCTGAGGAGGGTGGCAG	CGTGTTCTCATCCGTGGAGC
**XRCC2**	AAGATTCACCTGTGCATGGTGAT	ACATCGTGCTGTTAGGTGATAAAGC
**XRCC3**	CCGAGGTCTGGCACTTGCT	GCTGCAGTGCTGTAAGGATGC
**XRCC4**	GGACATCAAACAAGAAGGGGAA	GGATCTCGGTCAGCAGTCATTT

Supplemental Table 2: Pathway enrichment analysis of Reactome FI network modules of upregulated genes upon HSP90i by NW457 in LN229 cells.
Differentially upregulated genes upon HSP90i by NW457 (FDR < 0.1) were subjected to Reactome FI network construction with subsequent clustering and pathway enrichment analysis of modules with ≥ 9 genes. Top 3 hits per module are shown.

Module	Geneset	Number of proteins in geneset	Proteins from module	P-value	FDR	Nodes
0	AP-1 transcription factor network(N)	70	8	2.82E-08	1.20E-05	NR3C1, GJA1, EDN1, TP53, ATF3, HIF1A, CCL2, TRIP6
0	Glucocorticoid receptor regulatory network(N)	78	8	6.42E-08	1.36E-05	NR3C1, SMARCC2, IRF1, TP53, HDAC2, GATA3, SFN, CDK5R1
0	Direct p53 effectors(N)	137	9	3.89E-07	4.40E-05	SERPINE1, KAT2A, IRF5, TP53, ATF3, HDAC2, SFN, GADD45A, ARID3A
1	Integrin signalling pathway(P)	158	18	1.11E-16	2.68E-14	COL12A1, ITGB2, ACTG1, ITGA4, ACTN4, RHOB, RAP2B, COL4A2, COL4A1, RAPGEF1, VCL, COL11A1, MICALL1, FLNB, CAV1, ARHGAP26, COL1A2, COL5A1
1	Extracellular matrix organization(R)	262	18	4.44E-15	5.33E-13	COL12A1, ITGB2, PLOD1, F11R, COL27A1, ITGA4, PCOLCE, COL4A2, COL4A1, COL11A1, PDGFA, THBS1, SERPINH1, COL1A2, P4HA1, COL5A1, P4HA2, SDC1
1	Focal adhesion(K)	199	16	1.73E-14	1.39E-12	ACTG1, ITGA4, ACTN4, COL4A2, COL4A1, RAPGEF1, MYL9, VCL, PDGFA, THBS1, MYL12A, FLNB, CAV2, CAV1, COL1A2, ZYX
2	Mitotic Metaphase and Anaphase(R)	170	24	1.11E-16	4.00E-15	ZWILCH, BUB1B, CDC20, PSMD9, PPP1CC, XPO1, SPDL1, NUDC, ANAPC7, RANGAP1, DYNLL2, CKAP5, CDCA8, CENPA, AURKB, NUP43, BUB3, BUB1, UBE2C, NDE1, PLK1, NDC80, CENPE, CENPF
2	Signaling by Rho GTPases(R)	347	21	1.11E-16	4.00E-15	ZWILCH, BUB1B, CDC20, PPP1CC, XPO1, SPDL1, NUDC, RANGAP1, DYNLL2, CKAP5, CDCA8, CENPA, AURKB, NUP43, BUB3, BUB1, NDE1, PLK1, NDC80, CENPE, CENPF
2	Mitotic G2-G2/M phases(R)	177	17	1.11E-16	4.00E-15	CEP164, PSMD9, XPO1, NEK2, CKAP5, CCNA2, AJUBA, HMMR, AURKA, CCNB1, NDE1, PLK1, TPX2, NEDD1, CENPF, CDK1, CEP41
3	Spliceosome(K)	149	15	1.11E-16	4.55E-15	TCERG1, RBM25, DDX42, PRPF19, SNRNP70, AQR, PRPF3, SRSF5, SRSF6, SRSF7, SF3B3, SNU13, HSPA8, CHERP, XAB2
3	Processing of Capped Intron-Containing Pre-mRNA(R)	236	27	1.11E-16	4.55E-15	DDX42, PRPF19, ELAVL1, CCAR1, GLE1, SNRNP70, NDC1, SRRM2, AQR, SRRM1, HNRNPH1, PRPF3, SRSF5, SRSF6, SRSF7, SF1, SF3B3, CSTF3, SNU13, SYMPK, CPSF4, HSPA8, CPSF7, CHERP, XAB2, NUP35, RNPS1
3	mRNA surveillance pathway(K)	91	7	4.19E-08	1.13E-06	PNN, SRRM1, CSTF3, SYMPK, CPSF4, CPSF7, RNPS1
4	Cellular response to heat stress(R)	79	12	2.22E-16	4.62E-14	HSP90AB1, HSP90AA1, DNAJC2, DNAJC7, FKBP4, CRYAB, DNAJB1, HSPH1, BAG2, ST13, PTGES3, HSPA1B
4	HSP90 chaperone cycle for steroid hormone receptors (SHR)(R)	34	7	6.95E-11	7.23E-09	HSP90AB1, HSP90AA1, FKBP4, STIP1, PTGES3, DNAJA1, HSPA1B
4	Protein folding(R)	64	7	5.35E-09	3.69E-07	DCAF7, RGS7, CCT3, TCP1, CCT7, CCT4, CCT6A
5	Rap1 signaling pathway(K)	210	9	4.38E-10	1.23E-07	LPAR1, PDGFC, NGFR, VEGFC, PRKCA, MRAS, PLCB4, RAPGEF5, EPHA2
5	Ras signaling pathway(K)	232	8	2.61E-08	3.67E-06	RASSF1, PDGFC, NGFR, VEGFC, PRKCA, MRAS, RAPGEF5, EPHA2
5	PI3K-Akt signaling pathway(K)	354	8	6.47E-07	6.08E-05	IRS1, LPAR1, CREB3L1, PDGFC, NGFR, VEGFC, PRKCA, EPHA2
6	rRNA processing(R)	191	11	1.24E-14	2.98E-13	LTV1, HEATR1, NAT10, WDR43, RRP7A, PDCD11, NOP56, UTP4, NCL, TSR1, NOL11
6	Ribosome biogenesis in eukaryotes(K)	103	6	2.98E-08	3.58E-07	HEATR1, NAT10, WDR43, RRP7A, NOP56, UTP4
6	Aurora B signaling(N)	40	2	2.55E-03	0.0204	NSUN2, NCL

**7**	Class I MHC mediated antigen processing & presentation(R)	191	9	7.77E-16	2.56E-14	ZNRF1, DTX3L, FBXO17, RBBP6, LMO7, RBCK1, FBXO32, SKP2, FBXO22
**7**	Neddylation(R)	223	5	5.70E-07	9.12E-06	FBXO17, LMO7, FBXO32, SKP2, FBXO22
**7**	FoxO family signaling(N)	49	2	7.64E-04	8.40E-03	FBXO32, SKP2
**8**	Telomere Maintenance(R)	38	3	6.70E-07	1.07E-05	DKC1, RUVBL2, RUVBL1
**8**	Chromatin organization(R)	208	4	1.43E-06	1.14E-05	VPS72, RUVBL2, USP22, RUVBL1
**8**	C-MYC pathway(N)	25	2	6.74E-05	3.37E-04	RUVBL2, RUVBL1
**9**	RNA transport(K)	179	5	9.77E-08	1.08E-06	EIF4A2, PABPC4, EIF3J, EIF3E, EIF3B
**9**	RNA degradation(K)	79	4	2.50E-07	1.25E-06	CNOT6L, CNOT7, PABPC4, TOB2
**9**	Eukaryotic Translation Initiation(R)	112	4	9.97E-07	2.99E-06	EIF4A2, EIF3J, EIF3E, EIF3B

Supplemental Table 3: Pathway enrichment analysis of Reactome FI network modules of downregulated genes upon HSP90i by NW457 in LN229 cells. Differentially downregulated genes upon HSP90i by NW457 (FDR < 0.1) were subjected to Reactome FI network construction with subsequent clustering and pathway enrichment analysis of modules with ≥ 9 genes. Top 3 hits per module are shown.

Module	Geneset	Number of proteins in geneset	Proteins from module	P-value	FDR	Nodes
0	RNA Polymerase II Transcription(R)	911	35	8.88E-15	3.07E-12	RBPJ, CHEK1, JUNB, MAF, MAML3, KMT2E, KMT2C, TWIST1, SMYD2, SKIL, SMAD2, WWTR1, VDR, NR2F1, NFATC2, CDK6, BCL6, SP1, RARG, INTS13, SNAPC5, HEY1, EXO1, HEY2, KAT2B, RNMT, HDAC1, LEF1, HES1, POLR2L, ATAD2, RPRD2, HIPK2, RPA3, TAF7
0	Notch-mediated HES/HEY network(N)	47	8	8.73E-09	1.51E-06	RBPJ, TLE1, TWIST1, RCAN1, HEY1, HEY2, HDAC1, HES1
0	Notch signaling pathway(K)	53	8	2.19E-08	2.52E-06	RBPJ, MAML3, CTBP2, HEY1, HEY2, KAT2B, HDAC1, HES1
1	EGFR tyrosine kinase inhibitor resistance(K)	79	9	3.01E-11	1.19E-08	IGF1R, JAK2, IL6R, JAK1, PDGFRA, SHC4, TGFA, ERBB3, ERBB2
1	IGF1 pathway(N)	30	6	2.70E-09	5.35E-07	IRS2, IGF1R, GRB10, NCK2, PRKD1, PTPN11
1	Interferon-gamma signaling pathway(P)	15	5	4.95E-09	6.53E-07	JAK2, JAK1, IFNGR2, SOCS4, PTPN11
2	Mitotic G1 phase and G1/S transition(R)	134	14	2.22E-16	5.40E-14	MCM10, FBXO5, CCNE2, PSMD12, PSMD13, PSMA7, PSMB7, PSMB4, ORC1, CDC6, POLE4, PSMC5, PSMC3, PSMC2
2	DNA Replication Pre-Initiation(R)	85	12	1.11E-15	1.08E-13	MCM10, PSMD12, PSMD13, PSMA7, PSMB7, PSMB4, ORC1, CDC6, POLE4, PSMC5, PSMC3, PSMC2
2	Synthesis of DNA(R)	117	13	1.33E-15	1.08E-13	CCNE2, PSMD12, PSMD13, PSMA7, ANAPC11, PSMB7, PSMB4, ORC1, CDC6, POLE4, PSMC5, PSMC3, PSMC2
3	Extracellular matrix organization(R)	262	19	1.11E-16	7.66E-15	ITGB1, COL13A1, ITGB3, LAMA4, SDC3, TNC, NID2, COL19A1, SPP1, DAG1, ITGB8, ITGAV, ITGA2, MMP2, MMP15, COL8A1, ITGA6, COL9A3, CD44
3	ECM-receptor interaction(K)	88	13	1.11E-16	7.66E-15	ITGB1, ITGB3, LAMA4, TNC, SPP1, DAG1, ITGB8, ITGAV, ITGA2, ITGA6, COL9A3, FREM2, CD44
3	Integrin signalling pathway(P)	158	10	3.03E-12	1.29E-10	ITGB1, COL13A1, ITGB3, COL19A1, ITGB8, ITGAV, ITGA2, COL8A1, ITGA6, COL9A3
4	Class I PI3K signaling events mediated by Akt(N)	35	5	2.61E-08	5.43E-06	YWHAE, MAPKAP1, YWHAQ, YWHAZ, TBC1D4
4	mTOR signaling pathway(N)	64	5	5.08E-07	4.35E-05	YWHAE, MAPKAP1, YWHAQ, YWHAZ, DDIT4
4	Insulin-mediated glucose transport(N)	27	4	6.30E-07	4.35E-05	YWHAE, YWHAQ, YWHAZ, TBC1D4
5	Signaling by Rho GTPases(R)	347	21	1.11E-16	9.21E-15	RTKN, ARHGEF26, ROCK2, ARHGAP5, ARHGAP35, ARHGAP12, GNA13, FGD4, ARHGAP21, OPHN1, ARHGEF11, RHOC, MYO9A, CIT, TIAM2, MYL6, ARHGEF9, DEPDC7, FMNL2, RHOJ, TAX1BP3
5	G alpha (12/13) signalling events(R)	56	8	5.41E-13	2.22E-11	ARHGEF26, ROCK2, GNA13, FGD4, ARHGEF11, RHOC, TIAM2, ARHGEF9
5	p75 NTR receptor-mediated signalling(R)	88	6	4.54E-08	1.22E-06	ARHGEF26, GNA13, FGD4, ARHGEF11, TIAM2, ARHGEF9
6	Cholinergic synapse(K)	112	8	1.28E-10	1.32E-08	CHRM3, ITPR2, GNAI2, GNG2, GNG12, GNG11, GNB4, PLCB1
6	Serotonergic synapse(K)	114	8	1.47E-10	1.32E-08	ITPR2, GNAI2, HTR7, GNG2, GNG12, GNG11, GNB4, PLCB1
6	Glutamatergic synapse(K)	114	7	6.02E-09	2.73E-07	ITPR2, GNAI2, GNG2, GNG12, GNG11, GNB4, PLCB1

7	Graft-versus-host disease(K)	41	10	1.11E-16	1.22E-15	HLA-DMA, HLA-DMB, HLA-DOA, HLA-DQA1, HLA-DPA1, HLA-E, HLA-DPB1, HLA-DRA, HLA-DRB1, HLA-DQB1
7	Antigen processing and presentation(K)	78	13	1.11E-16	1.22E-15	CIITA, HLA-DMA, HLA-DMB, HLA-DOA, HLA-DQA1, HLA-DPA1, CD74, HLA-E, RFX5, HLA-DPB1, HLA-DRA, HLA-DRB1, HLA-DQB1
7	Asthma(K)	31	9	1.11E-16	1.22E-15	HLA-DMA, HLA-DMB, HLA-DOA, HLA-DQA1, HLA-DPA1, HLA-DPB1, HLA-DRA, HLA-DRB1, HLA-DQB1
8	Mitotic Prometaphase(R)	178	9	6.01E-12	4.15E-10	SSNA1, DSN1, PRKAR2B, RAD21, CLASP1, CLASP2, TUBB4B, STAG2, AKAP9
8	Mitotic G2-G2/M phases(R)	177	6	3.75E-07	9.52E-06	SSNA1, PRKAR2B, PHLDA1, CLASP1, TUBB4B, AKAP9
8	Cilium Assembly(R)	180	6	4.14E-07	9.52E-06	SSNA1, PRKAR2B, CEP97, CLASP1, TUBB4B, AKAP9
9	Circadian rhythm(K)	31	4	1.81E-07	5.31E-06	NR1D1, PER2, PER3, CRY1
9	Protein processing in endoplasmic reticulum(K)	166	6	1.83E-07	5.31E-06	ERO1B, HSPA5, EIF2AK4, DNAJC3, P4HB, NFE2L2
9	Circadian clock system(P)	8	3	3.02E-07	5.75E-06	PER2, PER3, CRY1
10	TBC/RABGAPs(R)	42	2	3.52E-04	7.76E-03	RAB4A, RAB6A
10	Pre-NOTCH Expression and Processing(R)	46	2	4.22E-04	7.76E-03	ATP2A2, RAB6A
10	Translocation of SLC2A4 (GLUT4) to the plasma membrane(R)	51	2	5.17E-04	7.76E-03	RAB4A, RAB14
